# Case Report: A prenatal case with sex discordance between non-invasive prenatal testing and fetal genetic testings due to maternal rare chromosome karyotype

**DOI:** 10.3389/fgene.2025.1546579

**Published:** 2025-05-06

**Authors:** Guoxing Zhong, Jiajia Wu, Zeyan Zhong, Dina Chen, Zhiyang Guan, Shaohui Huang, Jianhong Chen

**Affiliations:** Department of Medical Genetics and Prenatal Diagnosis, Huizhou First Maternal and Child Healthcare Hospital, Huizhou, Guangdong, China

**Keywords:** NIPT, cffDNA, case report, breakpoint, CNV-seq

## Abstract

**Background:**

Non-invasive prenatal testing (NIPT), which made use of cell-free DNA (cffDNA) in maternal blood, was currently being applied all over the world for the detection of common chromosome abnormalities. It had relatively high sensitivity and specificity. Nevertheless, studies demonstrated that false positive results happened in 0.3% of the cases due to several factors. These factors included confined placental mosaicism, maternal mosaicism, maternal transfusions, maternal malignancy, vanishing twins and maternal chromosomal abnormalities.

**Case report:**

We presented a case of a 27-year-old healthy woman, who had a high risk of trisomy 21 syndrome in first-trimester serum screening at 12 gestational weeks. The result of NIPT indicated a high risk of klinefelter syndrome (47, XXY) at 15 weeks gestation. Subsequently, amniocentesis revealed a normal female fetus karyotype (46, XX) at 18 weeks gestation. Discordant sex chromosome results emerged. Eventually, it was discovered that there was a rare maternal karyotype 46,X,der(X)t (X; Y) (p22.3; q11.2), which led to the sex discrepancy between the NIPT and the fetal prenatal diagnostic results.

**Conclusion:**

We presented a case in which there was a sex discrepancy between NIPT and fetal genetic testing due to a rare chromosome karyotype in the mother. NIPT was merely a prenatal screening test. Consequently, patients who had a screen-positive result for a chromosomal anomaly following NIPT ought to be properly counselled and advised to undergo an invasive diagnostic procedure for confirmation.

## Introduction

Non-invasive prenatal testing had been widely applied for prenatal screening of common fetal aneuploidies, such as trisomy 13, 18 and 21. The sensitivity and specificity of NIPT could be over 99% (Liao et al., 2014). However, 0.3% of the cases might been reported as false-positive NIPT results, owing to confined placental mosaicism (CPM), maternal mosaicism, maternal transfusions, a vanishing twin, maternal malignancy and maternal organ transplant (Samura and Okamoto, 2020; Balaguer et al., 2021). Here, we reported a case with sex discordance between NIPT and fetal genetic testings due to maternal rare chromosome karyotype.

## Case presentation

**FIGURE 1:**
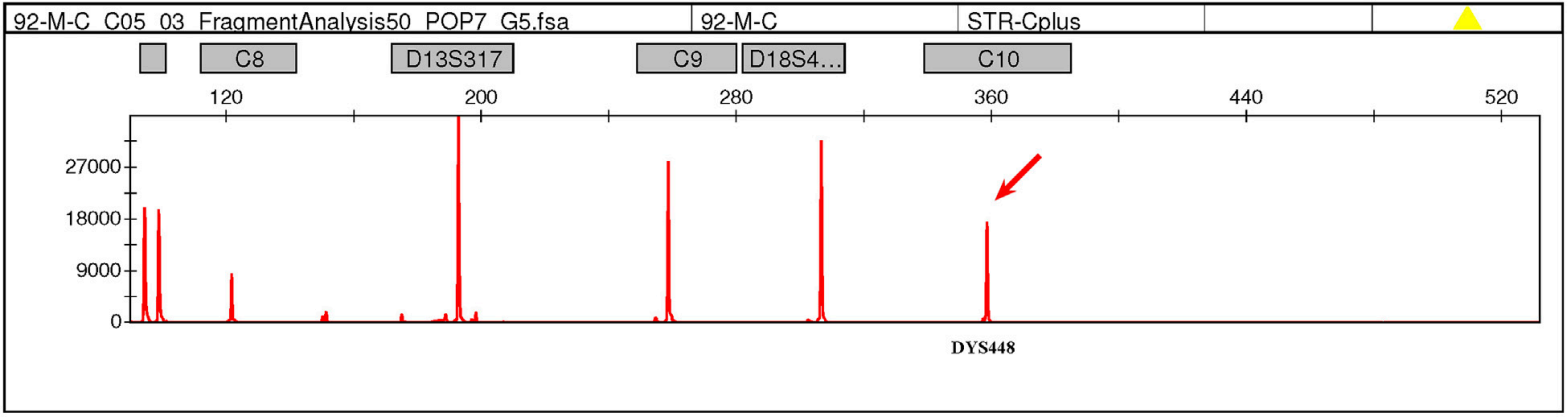
QF-PCR testing detected a signal at the DYS448 locus (Yq11.2) in the maternal peripheral blood, suggesting the possible presence of partial Yq11.2 fragment in the mother.

**FIGURE 2:**
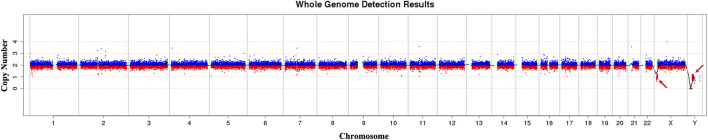
CNV-seq detected a 5.78 Mb deletion in the ChrX (p22.33-p22.31) region and a 8.46 Mb fragment duplication in the ChrY (q11.22) region in the maternal peripheral blood. (The detailed result was: seq [hg19] (1-22,X)×2, Yq11.221q11.223 × 1; seq [hg19] del(X) (p22.33-p22.31) ChrX:g.2710000_8490000del; seq [hg19] del(Y) (p11.31-q11.221) ChrY:g.2640000 _16020000del.).

**FIGURE 3:**
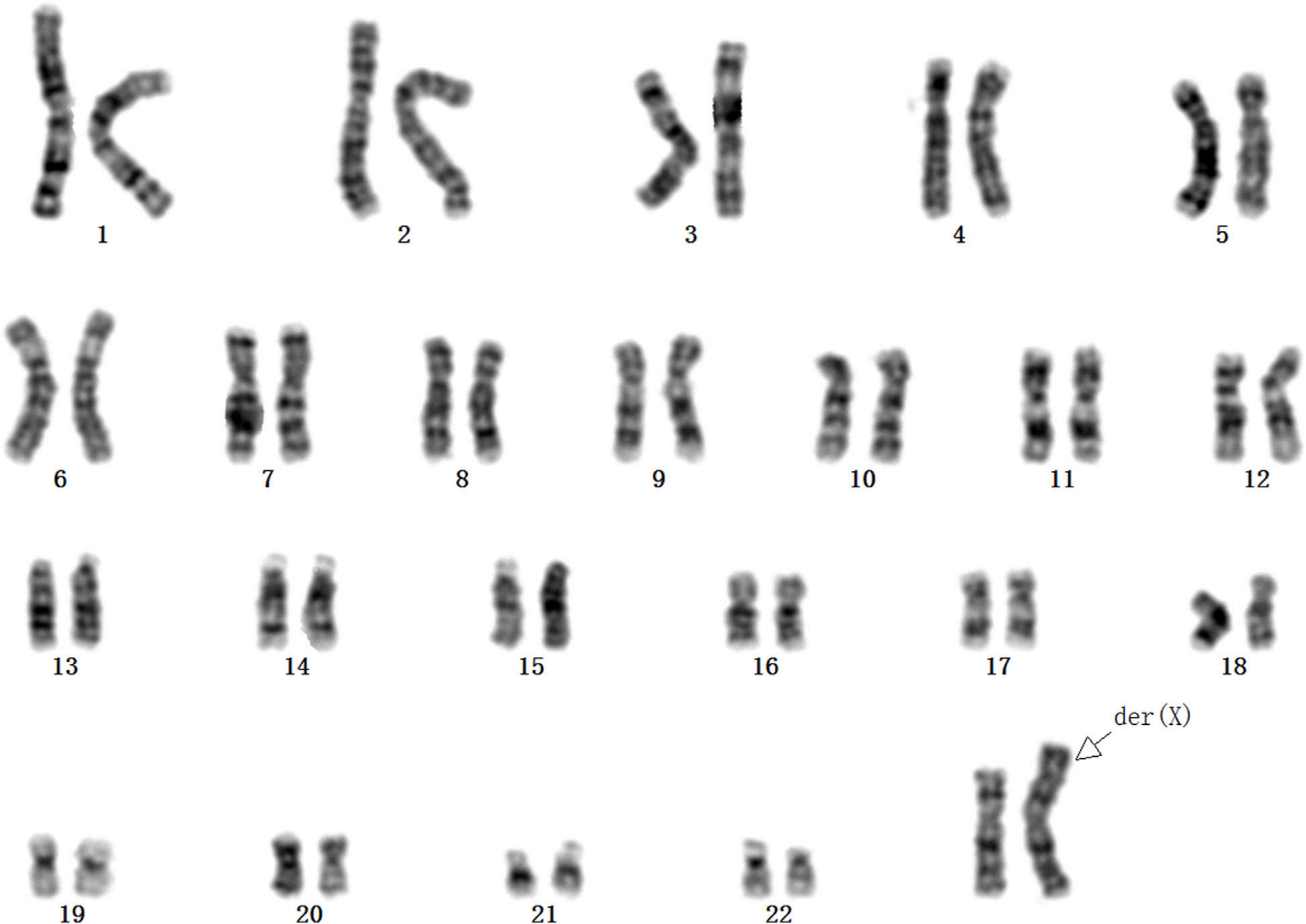
The karyotype for the maternal peripheral blood was 46,X,der(X)t (X; Y) (p22.3; q11.2).

This case was a 27-year-old, gravida 2, para 1, healthy woman. She delivered a healthy female infant at full term *via* vaginal birth with a birth weight of 2.15 kg in 2018. In 2023, first-trimester serum screening indicated that the fetus was at high risk of trisomy 21 syndrome (1/42), and nuchal translucency was 1.1 mm at 12 gestational weeks. The woman refused amniocentesis and requested NIPT testing at 15 weeks gestation. The result of NIPT indicated a high risk of klinefelter syndrome (47, XXY). Subsequently, amniocentesis was performed at 18 weeks gestation, quantitative fluorescent polymerase chain reaction (QF-PCR) analysis for aneuploid chromosomes 13, 18, 21, X and Y was negative, karyotype analysis and chromosomal microarray analysis (CMA) of the amniotic fluid revealed a normal female fetus karyotype (46, XX). Due to the inconsistency between NIPT and karyotype results, the genotype of the parents was further analyzed at 20 weeks gestation. Fatherly karyotype was normal (46, XY). However, QF-PCR testing detected a signal at the DYS448 locus (Yq11.2) in the maternal peripheral blood, suggesting the possible presence of partial Yq11.2 fragment in the mother (Figure 1). Besides that, a 5.78 Mb deletion in the ChrX (p22.33-p22.31) region and a 8.46 Mb fragment duplication in the ChrY (q11.22) region (Figure 2) was found in the maternal peripheral blood by copy number variation sequencing (CNV-seq) detection technology. Consistent with the previous two results, the karyotype for the maternal peripheral blood was 46,X,der(X)t (X; Y) (p22.3; q11.2) (Figure 3). Finally, the parents decided to continue the pregnancy because there were no significant abnormalities in the subsequent development of the fetus. A female infant was born at 38 weeks of gestation with a birth weight of 2.1 kg, karyotype analysis was normal, and no additional abnormalities were identified apart from the low birth weight.

## Discussion and conclusion

Previous case series reports had demonstrate that sex discordant results were detected in 1/1500-1/2000 of the pregnant women when they undertaked NIPT (Richardson et al., 2017). Maternal sex chromosome abnormality was one of the most important cause of false-positive result at NIPT (Chen et al., 2022). Here, we presented firstly a case of a rare maternal karyotype 46,X,der(X)t (X; Y) (p22.3; q11.2), which leaded to the sex discordance between NIPT and fetal prenatal diagnostic results. This pregnant woman did not show any obvious abnormal phenotype. And studies had shown that cases with the same karyotype as our case exhibited normal hormonal levels and fertility abilitiy (Huang et al., 2023). However, there were also studies show mild intellectual disability in adult (Spranger et al., 1997) or compounded ultrasound anomalies in the fetus (Cain et al., 2007) when they carried this rare karyotype. Translocations between the X and Y chromosomes might occur during paternal meiosis (Anik et al., 2013; Gao et al., 2013). Moreover, in light of the breakpoints and the magnitude of the translocated regions, such a crossover had the potential to result in diverse phenotypic consequences (Politi et al., 2024). Therefore, although these cases had the same karyotype, the different phenotypes might be due to different breakpoints. In addition, studies had shown that pathogenic copy number variations (CNVs) on X-derived chromosomes may also result in no apparent clinical phenotype due to X-chromosome inactivation (XCI) (Kim et al., 2019).

In summary, we presented a case where there was a sex discrepancy between NIPT and fetal genetic testing as a result of a rare chromosome karyotype in the mother. Maternal copy number variation, especially when she contained the Y chromosome fragment, was an important cause of false positive NIPT result for sex chromosomal aneuploidy. Furthermore, NIPT was just a screening test, not a diagnostic test. The cffDNA in maternal plasma came from apoptotic placental trophoblast cells. So it mostly had placental DNA. That meant the test result might not show the real fetal karyotype. Therefore, patients with a screen-positive result for a chromosomal anomaly subsequent to NIPT needed to be appropriately counselled and recommended to have an invasive diagnostic procedure for verification. This was crucial before any choices about the pregnancy were made.

## Data Availability

The original contributions presented in the study are included in the article/supplementary material, further inquiries can be directed to the corresponding author.
